# Giftedness: A Critical Analysis of Theories and Identification Methods in Light of Contemporary Neuroscience

**DOI:** 10.1002/jdn.70126

**Published:** 2026-04-21

**Authors:** Karin Reuwsaat, Bruno Poltronieri, Rogério Panizzutti, Bruna Velasques

**Affiliations:** ^1^ Institute of Biomedical Sciences Federal University of Rio de Janeiro Rio de Janeiro Brazil; ^2^ Institute of Psychiatry Federal University of Rio de Janeiro Rio de Janeiro Brazil; ^3^ Bioscience Department School of Physical Education of the Federal University of Rio de Janeiro (EEFD/UFRJ) Rio de Janeiro Brazil; ^4^ Federal Institute of Education, Science and Technology of Rio de Janeiro Rio de Janeiro Brazil

**Keywords:** cognitive neuroscience, giftedness, intelligence, neurodiversity

## Abstract

Giftedness is a complex and multifaceted construct that lacks clear conceptual consensus, resulting in challenges for standardizing identification criteria. Divergences between traditional psychometric models and contemporary developmental approaches create inconsistencies in assessment practices, especially when considering comorbidities with neurodevelopmental conditions such as ADHD, Autism Spectrum Disorder and Specific Learning Disorders. Central to this debate is whether giftedness should be defined solely by high IQ or expanded to include creativity, motivation and domain‐specific talents—or whether these represent separate but equally relevant constructs. Theoretical models such as the Three‐Ring Theory, Multiple Intelligences and the Triarchic Theory have broadened the scope of human potential. In parallel, Gagné's Differentiated Model and advances in cognitive neuroscience have revealed distinct neural patterns associated with general giftedness versus specific talents. This paper traces the epistemological evolution of giftedness from early psychometric paradigms to multidimensional frameworks, integrating neuroscientific evidence on structural, functional and cognitive correlates of high intellectual ability. Building on these insights, we propose the Differential Model of Giftedness and Talent, which conceptualizes giftedness as elevated cognitive potential, measurable through intellectual functioning, and talent as the developmental outcome emerging from the transformation of this potential into domain‐specific expertise. Motivation, creativity, engagement and persistence are highlighted as key mechanisms. This integrative framework provides a clearer conceptual basis for research, identification and educational interventions in gifted populations.

## Graphical abstract

This article reviews the evolution of giftedness from psychometric to multidimensional models and proposes the Differential Model of Giftedness and Talent, grounded in neuroscientific evidence distinguishing general giftedness from specific talents. Giftedness is defined as high cognitive potential, whereas talent reflects its developmental realization in domain‐specific expertise.

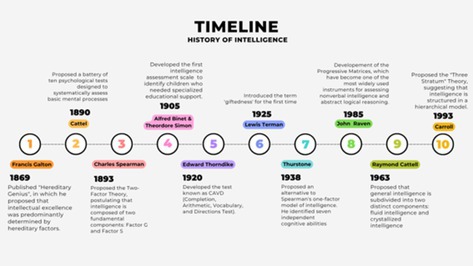

## 1 Introduction

Giftedness remains a construct without definitive consensus, presenting significant conceptual divergences between different theoretical currents and socio‐cultural contexts (Fernández et al. 2017; Desvaux et al. 2024). Such conceptual polysemy necessitates the formulation of robust operational definitions that can ensure validity and reliable identification (Robinson et al. 2000; Subotnik et al. 2011). The absence of unified criteria among leading theoretical models—from traditional psychometric approaches to more recent developmental perspectives—results in significant inconsistencies in identification processes. Terminological diversity includes giftedness, high intelligence, talent (Desvaux et al. 2024; Carman 2013; Ziegler and Raul 2000) and gifted, highly gifted or high intellectual potential (Vaivre‐Douret 2011).

Early detection is educationally crucial for cognitive and socio‐emotional development, requiring well‐defined identification criteria, particularly given the complexity of the differential diagnosis with neurodevelopmental disorders, especially ADHD (Attention Deficit Hyperactivity Disorder), ASD (Autism Spectrum Disorder) and Learning Disorder (Kontakou et al. 2022). Twice‐exceptionality concomitant giftedness and neurodevelopmental disorders present an additional challenge, requiring multidimensional assessment protocols to prevent diagnostic masking (Silverman and Gilman 2020; Lovett and Lewandowski 2006; Ruban 2005). Understanding the neurofunctional characteristics of gifted individuals is essential for diagnostic accuracy, precise prognoses and personalized therapeutic strategies.

Despite the growing interest in the subject, a significant gap remains in the integration of the epistemological evolution of giftedness with contemporary neuroscientific advances. Specifically, few studies systematically connect neurophysiological and neurostructural findings with theoretical frameworks and terminologies associated with giftedness. This conceptual fragmentation undermines the accuracy of unified diagnostic criteria development, cross‐study comparability and understanding of neural mechanisms underlying giftedness. Therefore, the present study aims to conduct a critical historiographical analysis of the epistemological evolution of the construct of giftedness, examining the paradigmatic shift from pioneering psychometric models to contemporary multidimensional models. Moreover, it aims to conduct an integrative synthesis of neuroscientific evidence on the neural correlates of giftedness, including structural, functional and cognitive markers.

## 2 Theoretical Foundations of Intelligence: Historical Evolution

The scientific study of giftedness traces its historical roots to Sir Francis Galton's (1822–1911) pioneering work. Influenced by Charles Darwin's evolutionary theory, Galton introduced a quantitative and hereditary approach to intelligence in *Hereditary Genius* (1869), proposing that intellectual excellence was largely inherited (Galton 1962). Despite ethical and scientific limitations, his work established foundations for psychometrics, including the concept of normal distribution and comparative measurement (Burt 1962).

In 1890, Cattell proposed a battery of psychological tests designed to systematically assess basic mental processes (e.g., reaction time, sensory discrimination, immediate memory and colour‐naming ability) (Cattell 1890). Although his paper did not explicitly use the term ‘intelligence’, it established fundamental principles for standardizing procedures and quantifying elementary mental functions, marking the transition from Galton's genealogical studies to experimental psychometrics proper.

Spearman subsequently proposed the Two‐Factor Theory, postulating that intelligence comprises Factor G (general intelligence, inherent in all cognitive tasks) and Factor S (specific intelligence related to particular abilities). Spearman demonstrated that performance across different cognitive tasks showed moderate to high correlations, supporting the existence of a general intelligence factor (Jensen 1993; Dolan 2000).

In 1905, Alfred Binet and Theodore Simon, in France, developed the first intelligence assessment scale to identify children who needed specialized educational support. The test measured judgement, common sense, initiative and adaptability, focusing on the individual's ability to integrate into the social environment, rather than their academic performance (Binet and Simon 1905).

Thorndike criticized intelligence tests for their linear assessment of intellectual capacity, which overlooked the complexity of multiple individual abilities and interpersonal influences (Thorndike 1920). In response, he developed the CAVD test (Completion, Arithmetic, Vocabulary and Directions Test), measuring intellectual level through specific subtests targeting distinct abilities (Thorndike 1927). This multifactorial approach significantly influenced the Wechsler Scales—Wechsler (WAIS and WISC), whose underlying theory conceptualizes intelligence as a set of diverse and interdependent mental abilities rather than a single general factor (Canivez et al. 2019).

In 1921, Terman revised Binet's scale to create the Stanford–Binet Scale, one of the most widely used intelligence tests. In 1925, Terman introduced the term ‘giftedness’ for the first time, seeking to deconstruct negative stereotypes that associated this condition with social and psychological problems. His longitudinal study of 1528 individuals over several decades indicated that gifted children generally demonstrated advanced physical development, satisfactory social adjustment and psychological stability (Terman 1925).

Thurstone (1938) proposed an alternative to Spearman's one‐factor model, identifying seven independent cognitive abilities: verbal fluency, verbal comprehension, spatial visualization, numerical facility, associative memory, reasoning and perceptual speed. However, when he tested intellectually heterogeneous children, these abilities were not wholly independent, revealing evidence of an underlying general factor. Thurstone integrated the general factor (g) with the seven specific abilities—laying the foundation for hierarchical theories and later models of multiple intelligences (Gardner 1983; Sternberg 1985).

In 1938, Raven developed Raven's Progressive Matrices, an instrument for assessing non‐verbal intelligence and abstract logical reasoning. The test requires identifying underlying logic to complete incomplete visual patterns, measuring fundamental cognitive abilities such as abstract reasoning, pattern perception and non‐verbal problem‐solving—core components of general intelligence (Raven et al. 2003). Its broad applicability across clinical, educational and research settings stems from its cross‐cultural validity and equitable assessment regardless of cultural or socio‐economic differences (Carpenter et al. 1990).

Cattell proposed in 1963 that general intelligence (g) comprises two distinct components: fluid intelligence (fg), the ability to solve new reasoning problems (Unsworth et al. 2014), and crystallized intelligence (cg), the application of previously acquired knowledge and skills (Cattell et al. 1941; Cattell 1963). The distinction between fluid and crystallized intelligence provides a more detailed understanding of the structure of human intelligence, highlighting the interaction between basic cognitive processes and acquired knowledge, which is relevant for studies in cognitive psychology and psychometrics (Brown 2016).

In 1993, Carroll proposed the ‘Three Stratum’ (3S) Theory, a hierarchical model of intelligence comprising three levels: Stratum III (general intelligence, or g factor), Stratum II (eight broad abilities, including fluid intelligence, crystallized intelligence, memory and learning, visual perception, auditory perception, retrieval capacity, cognitive speed and processing speed) and Stratum I (over 70 specific abilities, such as verbal reasoning and short‐term memory). Developed through extensive factor analysis, this model became one of the most comprehensive in psychometrics (McGrew 2009). Carroll and Horn–Cattell theories, while both adopting a hierarchical approach to the structure of intelligence, differ in a key aspect: Carroll accepts the existence of a general intelligence factor (g) at the top of the hierarchy, whereas Horn–Cattell rejects a single overarching factor, focusing instead on broad cognitive abilities such as fluid and crystallized intelligence. Thus, in 1997, McGrew (2009) synthesized these two important perspectives, giving rise to what later became known as the Cattell–Horn–Carroll (CHC) model. This integrated model is considered the most widely recognized psychometric taxonomy of cognitive abilities (McGrew 2009; McGill and Dombrowski 2019; Wasserman 2019). The CHC model was later updated, proposing 16 broad cognitive skills (Fluid Intelligence, General Knowledge, Quantitative Knowledge, Reading‐Writing, Crystallized Intelligence, Short‐Term Memory, Long‐Term Storage and Retrieval, Visual Processing, Auditory Processing, Olfactory Skills, Tactile Skills, Psychomotor Skills, kinaesthetic processing, Processing Speed, Reaction Speed and Psychomotor Speed), which are subdivided into more than 80 specific skills (Schneider and McGrew 2012).

Over the course of more than a century of scientific research on intelligence, a universal consensus on its definition has yet to be established (Neisser et al. 1996). This disagreement stems from the broad and multifaceted nature of the construct, which encompasses cognitive, social and cultural dimensions. However, one of the most widely adopted concepts in the literature defines intelligence as the general ability to reason, plan, solve problems, think abstractly, assimilate complex concepts, learn quickly and adapt to new experiences. This ability transcends academic knowledge or mere intellectual dexterity, reflecting instead a comprehensive ability to understand the environment—capturing contextual nuances, assigning meaning to information and determining appropriate actions (Gottfredson 1997; Prabakaran 2025).

**FIGURE 1:**
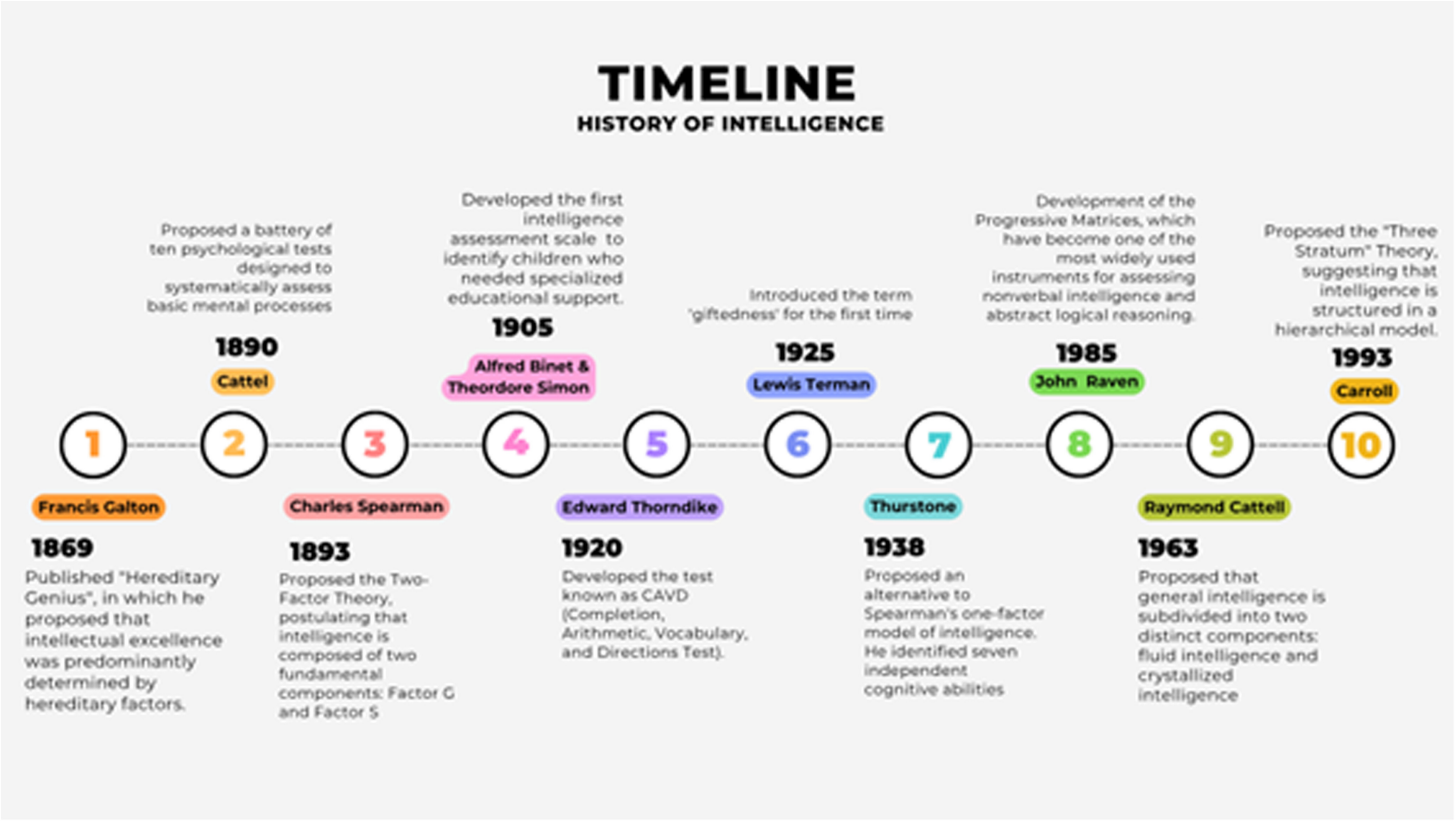
Summary timeline of theories of intelligence.

Figure 1 summarizes this historical evolution through a timeline that highlights the main scholars in the field of intelligence.

## 3 Theoretical Foundations of Giftedness: Historical Evolution

Terman was the first author to introduce the term ‘giftedness’, defining gifted children as those in the top 1% of the school population, with an intelligence quotient (IQ) of 140 or above (Terman 1925). His longitudinal study revealed accelerated physical development, adequate social adjustment and psychological stability, emphasizing the need for suitable educational environments with curricular adjustments to accommodate faster learning (Jolly 2008).

In the 1970s, Renzulli proposed the Three‐Ring Theory of Giftedness, defining giftedness as the dynamic intersection of three characteristics: above‐average ability (general or specific ability), creativity and task commitment (Renzulli 2012). This approach diverges from the traditional view that giftedness is exclusively associated with high IQ, highlighting that individuals can demonstrate exceptional abilities in specific areas, such as ballet, mathematics, musical composition or sports, without necessarily presenting above‐average performance in conventional intelligence tests (Renzulli 2012; Renzulli 1978).

Despite its relevance, the Three‐Ring Theory has faced criticism for lacking scientific rigour because of subjectivity in assessing creativity and task commitment, which vary across cultural contexts and challenge empirical testing (Sternberg 2000). Although general abilities can be measured objectively, domains like ballet rely on subjective evaluations, complicating replication (Renzulli 1978; Beghetto and Kaufman 2007). Nevertheless, the theory remains central in gifted education, emphasizing multifaceted talent development. Renzulli (2012) conceptualizes giftedness as a dynamic process shaped by opportunities and context—a perspective that has significantly influenced educational practices and public policies for identifying and developing talents.

In 1983, Gagné proposed the Differentiated Model of Giftedness and Talent (DMGT), distinguishing giftedness (natural potential or innate abilities) from talent (exceptional performance through learning and practice) (Gagné 2004). Talents emerge progressively as these aptitudes are systematically developed into competencies across specific fields such as academics, arts, business, leisure, social affairs, sports and technology (Gagné 2004). Gagné also emphasizes intrapersonal catalysts (motivation, health, self‐management and personality) and environmental catalysts (cultural, family and social contexts) that can positively or negatively influence talent development. Although widely recognized for its detailed and multifaceted framework, critics argue that the DMGT's complexity hinders its practical application in educational settings that require clear, objective identification criteria (Ziegler and Raul 2000). Additionally, the emphasis on catalysts may underestimate the role of natural abilities, potentially minimizing the importance of innate potential.

In (Gardner 1983), Gardner proposed the Theory of Multiple Intelligences, arguing that intelligence is not a unitary construct but a set of relatively independent cognitive abilities that can develop uniquely in each individual. Gardner argues that multiple intelligences are shaped by biological, cultural and contextual factors, allowing for a more inclusive and diverse approach to education and human development. Critics cite a lack of empirical evidence supporting the independence and validity of proposed intelligences, as well as the absence of a clear structure for their interaction, which limits their practical application (Waterhouse 2006; Sternberg 1985).

Sternberg (1985) proposed the Triarchic Theory of Intelligence (TTI), where intelligence is composed of three interrelated elements: analytical (ability to solve problems and evaluate ideas), creative (ability to generate new ideas and adapt to unexpected situations) and practical (ability to apply knowledge effectively in real contexts) (Sternberg 1985). Sternberg proposes that giftedness should be understood as a dynamic process of developing expertise (a set of skills necessary for a high level of mastery in one or more domains), where motivation, effort and environment play crucial roles, instead of being seen as a static or innate attribute (Sternberg 1985; Sternberg 2000). Both theories value creativity, though Sternberg focuses on general intelligence whereas Renzulli targets gifted identification. A key criticism is the difficulty of operationalizing and measuring the three components.

The theoretical contributions of Renzulli (1978), Sternberg (2000) and Gardner (1999) significantly reconfigured the construct of giftedness in the educational context, transcending traditional psychometric models by incorporating dimensions that IQ instruments cannot measure. These models converge in three fundamental aspects: (1) the need for multifactorial identification systems that contemplate specific skills (Reis and Renzulli 2010); (2) the development of differentiated pedagogical interventions based on distinct cognitive profiles (Sternberg 2000; Reis and Renzulli 2010); and (3) the recognition of the determining influence of the socio‐cultural context in the development of potential (Gardner 1983), refuting strictly innate conceptions. However, these approaches lack greater methodological robustness, particularly about to the operationalization of constructs and the predictive validity of their models (Waterhouse 2006; Lubinski 2004). In this scenario, the differentiation proposed by Gagné in his DMGT (Gagné 2004) stands out, which establishes a crucial epistemological distinction between giftedness (as an innate biopsychological potential) and talent (as a competence developed through systematic practice), offering a more parsimonious theoretical framework for research and practical intervention. In addition, the socio‐cultural perspective proposed by Barab and Plucker (2002) emphasizes that talent emerges from talented transactions between the individual and the environment, arguing that no one possesses talent in a static way, but rather that everyone has the potential to engage in talent development processes when ecological conditions are favourable.

Theoretical models of giftedness reflect distinct epistemological foundations. Terman's (1925) positivist, psychometric approach conceptualizes intelligence as a stable, measurable trait, supported by longitudinal evidence of IQ stability across the lifespan. In contrast, Renzulli's Three‐Ring Conception (1978, 2012) adopts a multifactorial, interactionist perspective, expanding the construct to include creativity and task commitment—dimensions characterized by greater contextual variability and lower psychometric precision. Gagné (2004) introduces an ontological distinction between innate potential (giftedness) and systematically developed skills (talent), differentiating natural predisposition from performance acquired through practice and environmental mediation. Gardner (1983, 1999) proposes a pluralistic epistemology rejecting unitary intelligence, whereas Sternberg (1985, 2000) advances an integrative, contextual approach emphasizing adaptation and expertise development.

Motivation and creativity assume distinct functional roles across models: structural components of identification for Renzulli, developmental catalysts for Gagné and constitutive dimensions of adaptive intelligence for Sternberg—serving epistemologically different purposes despite their shared presence.

**FIGURE 2:**
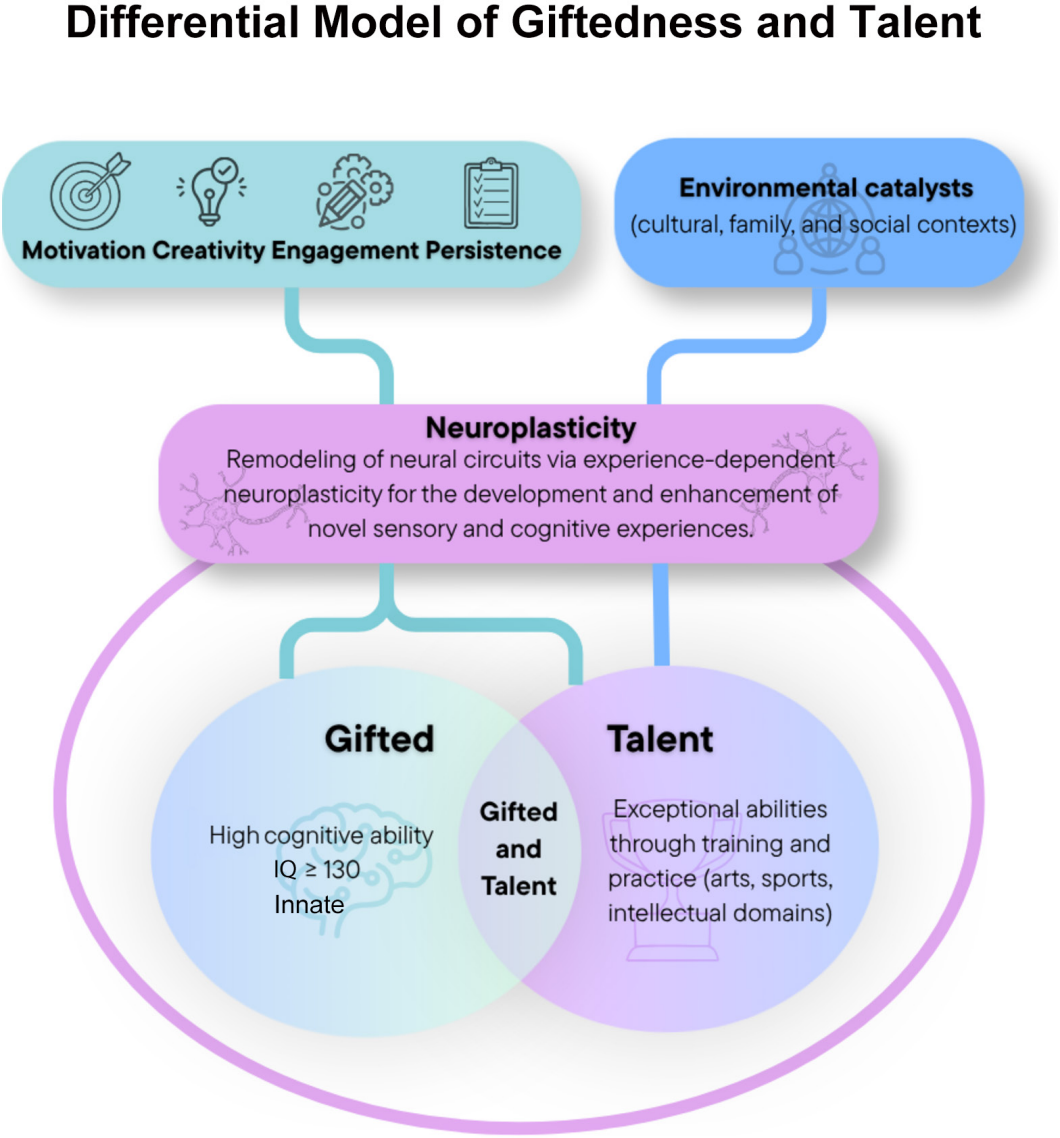
Schematic representation of the Differential Model of Giftedness and Talent—The model distinguishes two central constructs: giftedness, defined as the possession of innate superior cognitive abilities (e.g., IQ ≥ 130, indicative of high intellectual capacity), and talent, characterized by the systematic development of exceptional abilities through training and deliberate practice in specific domains (arts, sports and intellectual fields). Both share predictors such as motivation, creativity, engagement and persistence, but talent additionally requires environmental catalysts (cultural, family and social contexts) for its full manifestation. The neurobiological basis of this process is neuroplasticity, defined as the remodelling of neural circuits mediated by experience, allowing the enhancement of sensory and cognitive functions. This mechanism supports the idea that giftedness is associated with the refinement of existing neural networks, whereas talent is built throughout development through the interaction between genetic potential and intentional practice.

Building on the theoretical developments discussed above and considering recent evidence indicating that current research still insufficiently explains how psychological constructs such as motivation, creativity and personality traits shape the manifestation of giftedness (Kuznetsova et al. 2024; Sternberg et al. 2022), this study proposes an integrative framework termed the Differential Model of Giftedness and Talent (Figure 2). The model aims to increase conceptual precision in the field of high abilities by clarifying the distinction between giftedness as elevated cognitive potential and talent as the development of exceptional performance in specific domains, while acknowledging the developmental interdependence between these constructs. Within this framework, giftedness is conceptualized as a latent biopsychological potential predominantly associated with high levels of general cognitive functioning, particularly fluid and/or crystallized intelligence, typically observable in the upper percentiles of intellectual performance (e.g., IQ ≥ 130). This potential is related to advanced development of cognitive processes such as memory, logical reasoning, attentional control, visuospatial processing and language abilities. In contrast, talent is understood as a developmental outcome that emerges through the progressive transformation of this potential into high‐level performance in specific domains—including academic, artistic or athletic contexts—through the interaction of deliberate practice, creativity, motivation and favourable environmental conditions across extended learning trajectories.

The central contribution of the model lies in clarifying the functional role of psychological variables frequently cited in giftedness theories but not consistently operationalized across them. In this framework, motivation is conceptualized as a dispositional characteristic associated with intellectual curiosity and engagement in individuals with gifted potential, whereas in talent development, it functions as a catalytic factor sustaining deliberate practice and expertise acquisition. Creativity is understood as a mechanism through which cognitive potential is transformed into meaningful and domain‐relevant production, supporting originality and cognitive flexibility. Engagement and persistence further contribute to the consolidation of expertise over time. From a theoretical and epistemological perspective, the Differential Model of Giftedness and Talent integrates key elements from psychometric, developmental and socio‐cultural approaches while addressing a critical gap in the literature: the need to clarify how cognitive potential, psychological processes and contextual factors interact in the emergence of high‐level performance. By articulating these dimensions within a single conceptual framework, the model contributes to reducing terminological ambiguity and provides a more coherent basis for future research, identification practices and educational interventions in gifted and high‐ability populations.

Although the Differential Model of Giftedness and Talent is conceptually rooted in Gagné's DMGT, the two frameworks differ in key theoretical and operational aspects. In the DMGT, giftedness refers to natural abilities distributed across multiple domains—intellectual, creative, socioaffective and sensorimotor—whereas talent reflects the systematic development of these aptitudes into competencies through learning and practice (Gagné 2004). In contrast, the Differential Model adopts a more psychometrically oriented definition of giftedness, conceptualizing it primarily as elevated cognitive potential associated with high levels of general intellectual functioning, typically identified through standardized intelligence measures (e.g., IQ ≥ 130). The Differential Model adopts a more parsimonious approach informed partly by Renzulli's theoretical contributions, emphasizing motivation and persistence as central mechanisms supporting both the expression of gifted potential and the long‐term development of talent. Thus, while maintaining continuity with Gagné's developmental perspective, the Differential Model seeks to address persistent conceptual ambiguities in the field by prioritizing clearer operational definitions and greater psychometric applicability in the identification of high‐ability individuals.

## 4 Evidence on Giftedness in Light of Neuroscience

Research in the neuroscience of giftedness predominantly identifies gifted individuals through validated psychometric instruments, particularly the Wechsler Intelligence Scales and Raven's Progressive Matrices (Kuznetsova et al. 2024). Longitudinal studies, such as that conducted by Deary et al. (2000), demonstrate consistency in IQ scores across the lifespan, with a correlation coefficient of 0.63 between assessments performed at ages 11 and 79. This finding supports the hypothesis of the relative invariability of the intellectual construct throughout the life cycle.

However, most studies on giftedness focus on children and adolescents—a period marked by significant neurodevelopment maturation, which involves morphological transformations in the central nervous system (Shaw 2007; Shaw et al. 2006). During functional neurodevelopment, cortical grey matter maturation follows distinct temporal trajectories across brain regions. Longitudinal neuroimaging studies indicate that grey matter volume peaks around age 12 in the frontal and parietal lobes and around age 16 in the temporal lobe and continues to develop in the occipital lobe until approximately age 20 (Giedd et al. 1999). These changes are associated with the process of neural pruning—an extensive mechanism by which a large number of initially overproduced axons, dendrites and synapses are progressively eliminated throughout development in the nervous system. Concurrently, the remaining synapses are preserved, strengthened and refined, contributing to the efficiency and specialization of neural networks (Faust et al. 2021). These considerations are fundamental and will be discussed in the following sections.

### 4.1 Structural Brain Changes Related to Giftedness

Intelligence has been associated with a distributed neural network encompassing regions such as the dorsolateral prefrontal cortex, parietal lobe, anterior cingulate cortex and specific areas of the temporal and occipital lobes (Jung and Haier 2007). White matter, in turn, mediates communication between these regions, indicating that high intelligence requires efficient information transfer across distributed neural networks (Deary et al. 2010; Stammen et al. 2023). Three principal hypotheses have been proposed to explain the association between higher fractional anisotropy values and elevated intelligence: (i) faster information processing due to increased myelination; (ii) more direct processing resulting from parallel and homogeneous fibre orientation; and (iii) greater parallel processing associated with higher axonal density. Collectively, these findings suggest that individuals with higher intelligence scores tend to exhibit more pronounced anisotropic diffusion patterns in white matter (Stammen et al. 2023). As summarized by Genç and Fraenz (2021), global white matter integrity plays a crucial role in efficient information transfer and, consequently, intellectual performance, given that intelligence emerges from a dynamic system composed of interacting subcomponents (Barbey 2018).

The prefrontal cortex has consistently been linked to intelligence (Jung and Haier 2007; Deary et al. 2010; Basten et al. 2015), playing a central role in complex cognitive abilities, including abstract reasoning, problem‐solving, memory retrieval, attention, working memory, social cognition, language and planning (Cabeza and Nyberg 2000; Wood and Grafman 2003). In gifted children, integration between the hippocampus and putamen and the prefrontal cortex via highly interconnected white matter tracts is enhanced, favouring superior memory capacity and learning abilities (Kuhn et al. 2021). Supporting these findings, graph‐theory–based analyses have shown that gifted children exhibit more globally integrated and versatile network topologies, suggesting a distinct brain organization (Solé‐Casals et al. 2019; Khundrakpam et al. 2016; Li et al. 2009).

Mathematically gifted individuals demonstrate distinctive patterns of brain organization, including increased intrahemispheric connectivity associated with complex mathematical reasoning (Singh and O'Boyle 2004; O'Boyle et al. 2005; Prescott et al. 2010). They also demonstrate more focal and economical brain activation during complex mathematical problem‐solving (Zhang et al. 2015), although neural efficiency is modulated by task demands and domain‐specific expertise rather than representing a universal marker of giftedness (Waisman et al. 2023). Morphometric studies further report variations in cortical thickness within the default mode and frontoparietal networks in mathematically gifted adolescents (Navas‐Sánchez et al. 2016), as well as greater efficiency in long‐range information integration across anatomically distant regions in gifted children (Ma et al. 2017), suggesting that differentiated cortical architecture and network organization underpin superior mathematical and higher order cognitive performance.

Beyond connectivity, evidence suggests a positive correlation between IQ and grey matter volume in the frontal, parietal, temporal and occipital lobes in adults (Haier et al. 2009; Haier et al. 2004). This association is also observed during neurodevelopment, between ages 5 and 18, with significant correlations between global grey matter volume and IQ (*R* = 0.90, *p* < 0.001) (Wilke et al. 2003). IQ accounts for approximately 9% (Wilke et al. 2003) to 15% (Reiss et al. 1996) of the variance in grey matter volume.

Thus, morphometric features of the cerebral cortex, such as cortical thickness and surface area, emerge as important predictors of cognitive ability. Studies indicate that higher intelligence levels are associated with reduced cortical thickness and increased cortical surface area (Feczko et al. 2009; Panizzon et al. 2009), reflecting variations in microstructural organization, such as neuronal density (Mountcastle 1997). These variations may be attributed to neurobiological processes such as synaptic pruning (Huttenlocher and Dabholkar 1997; Petanjek et al. 2011) and myelination (Gogtay and Thompson 2010), which modulate neural efficiency during childhood.

### 4.2 Functional Brain Changes Related to Giftedness

Event‐related potentials (ERPs) are sensitive indices of real‐time brain neural processing (Duan and Shi 2014). The P300 component reflects attentional resources allocated (Scisco et al. 2008) and processing speed (Hillman et al. 2006; Dibbets and Jolles 2006; Folstein and Van Petten 2008). Studies have shown that gifted children demonstrate faster and more accurate performance, accompanied by higher P300 amplitudes and/or shorter latencies across cognitive tasks (Kranzler et al. 1994; Duan and Shi 2014; Zhang et al. 2007; Duan et al. 2009; De Pascalis et al. 2008; Neubauer and Fink 2009). However, lower P300 amplitudes in highly intelligent individuals during complex tasks may reflect more efficient allocation of neural resources, extending the neural efficiency hypothesis (Jia et al. 2023). Similarly, enhanced N2 amplitudes and shorter latencies indicate more efficient conflict monitoring in gifted children (Liu et al. 2011; Li et al. 2020), a pattern corroborated by a systematic review highlighting stronger late ERP components and top‐down engagement in gifted groups (Kuznetsova et al. 2024). Even under resting‐state conditions, in the absence of explicit cognitive tasks, gifted individuals demonstrate more intense and complex brain activity (Lutzenberger et al. 1992), suggesting an elevated state of cognitive readiness. In mathematically gifted adolescents, resting‐state EEG reveals a pattern of relative right‐hemispheric inactivity characterized by increased alpha power, suggesting that these regions remain in a state of readiness that enables more efficient activation through sharper alpha desynchronization during cognitive engagement (Alexander et al. 1996). This distinctive resting‐state pattern aligns with broader evidence that the maturational trajectory of neural activity in these individuals parallels that of adults, as their alpha rhythms resemble those of university‐level populations—indicating early neurocognitive maturation that persists or enhances into adulthood (Alexander et al. 1996). Corroborating these EEG findings, fMRI studies demonstrate similar BOLD signals between gifted adolescents and adults (O'Boyle et al. 2005). Furthermore, gamma rhythm (30–45 Hz) shows task‐dependent modulation: Gifted adolescents exhibit lower power during simple reasoning tasks—consistent with the neural efficiency hypothesis—and increased power as task complexity rises (Zhang et al. 2015).

Waisman et al. (2025) found that mathematically talented individuals exhibit focal, efficient parieto‐occipital ERPs during insight problem‐solving, indicating neural specialization, whereas generally gifted individuals show diffuse activation patterns reflecting broader network engagement. These findings support partially dissociable neurocognitive bases for general intelligence and mathematical expertise.

### 4.3 Dynamic Brain Measures Related to Giftedness

Functional neuroimaging data support the hypothesis that more intelligent brains process information more efficiently—that is, with reduced neural resource recruitment during cognitive tasks—provided that the task complexity is sufficient to discriminate among different levels of intelligence without exceeding the cognitive capacity of gifted individuals (Haier et al. 1988). In contrast, under high task difficulty, gifted individuals tend to exhibit greater neural activation. In contrast, individuals with lower cognitive capacity are more likely to disengage, resulting in a positive correlation between neural effort and intelligence (Neubauer and Fink 2009).

The neural efficiency hypothesis thus suggests that individuals with higher IQs utilize cortical resources more efficiently during low‐demand tasks, whereas more complex cognitive demands require increased cerebral recruitment (Neubauer and Fink 2009). Neural efficiency has been linked to both quantitative and qualitative features of brain networks, including increased grey and white matter density, earlier brain maturation, prolonged myelination, high structural and functional interconnectivity and elevated interhemispheric activation (Neubauer and Fink 2009; Haier et al. 1988). These characteristics are associated with superior intellectual functioning, including faster information processing, reduced energy consumption, enhanced executive efficiency and greater proficiency in analogical, abstract and creative reasoning (Neubauer and Fink 2009; Lutzenberger et al. 1992). A systematic review by Kuznetsova et al. (2024) corroborates these findings, demonstrating that gifted children show superior performance in verbal working memory, inhibition, geometric problem‐solving, attentional switching and elementary information processing, alongside amplified and accelerated brain activity during complex cognitive processes.

Network neuroscience reinforces this view, asserting that brain systems with higher global structural and functional efficiency are consistently associated with higher general intelligence scores in both children and adults (Ng et al. 2024). An innovative study by Wilcox et al. (2026) on the network architecture of general intelligence (g factor) within the human connectome, grounded in Network Neuroscience Theory (NNT), challenges localizationist models by demonstrating that intelligence emerges from distributed systemic properties, including multiple networks, weak long‐range connections, modal control regions and small‐world topology. The findings, replicated in an independent sample of 831 participants from the Human Connectome Project, evidence the predictive power of joint structural‐functional modelling and emphasize the transition from region‐specific approaches to understanding global brain dynamics in intelligence.

Although highly intelligent individuals may develop multiple areas of expertise, most achieve specialized competence in one domain through deliberate effort and sustained practice rather than as an automatic consequence of intelligence (Koziol et al. 2010). Studies on specific talents—for example, in musicians—have demonstrated distinctive patterns of neuroplasticity, with significant structural reorganizations in motor, auditory and visuospatial brain regions (Petanjek et al. 2011), supporting the crucial role of deliberate practice in the development of expertise (Ericsson et al. 2009).

This dissociation between giftedness and talent is also observed in mathematically talented individuals, who show greater brain activation during highly specialized tasks. This phenomenon temporarily contradicts the neural efficiency principle typically observed in gifted individuals (Neubauer and Fink 2009). Waisman et al. (2025) investigated this dissociation in the mathematical domain, demonstrating that success in insight problem‐solving correlates strongly with general giftedness, whereas mathematical expertise is essential for strategy‐based problem‐solving. Additionally, the authors identified greater ERP amplitude at PO4/8 electrodes during insight resolution processes, suggesting distinct neurophysiological markers for different forms of mathematical ability.

Current evidence thus challenges traditional theoretical models by revealing distinct neural patterns between general giftedness—associated with greater global efficiency and cognitive network integration (Gómez León 2020)—and specific talents, which emerge from more localized cerebral specialization (Subotnik et al. 2011). These findings imply separate neurodevelopmental trajectories with unique neural and cognitive mechanisms, indicating that giftedness and specific talents represent distinct manifestations of brain development rather than equivalent phenomena.

## 5 Conclusion

Giftedness is a complex, multifaceted condition that requires an interdisciplinary approach integrating psychometrics, neuroscience and education. Despite advances in understanding its neurobiological bases and theoretical models, important gaps still require further research. Among current challenges is the need to clarify the neurophysiological mechanisms underlying both giftedness and specific talents, as well as to improve identification criteria to prevent misclassification, especially in cases of twice exceptionality, where characteristics of giftedness may overlap with conditions such as ADHD or ASD.

A key challenge is identifying biomarkers associated with giftedness to complement traditional assessments and improve early identification. There is also a lack of validated instruments to measure constructs such as creativity and motivation, despite their relevance in theoretical models. These gaps underscore the need for studies linking psychometric measures with neurophysiological indicators, such as EEG, fMRI and ERPs, to strengthen the evidence base and refine the understanding of giftedness.

Current evidence indicates that intellectual giftedness is associated with (1) greater white matter integrity and connectivity; (2) more integrated and less segregated network topology; (3) volumetric increases in subcortical structures linked to explicit memory; (4) partial dissociation between general giftedness and mathematical talent; (5) positive correlations between IQ and grey matter volume; (6) ERP differences (P300 and N2) reflecting enhanced attentional efficiency and faster processing; and (7) distinctive patterns of electrical activity at rest and during cognitive tasks. Nevertheless, methodological heterogeneity—including divergent definitions of giftedness, variability in age ranges and differences in experimental paradigms—continues to limit comparability across studies and constrains the formulation of a unified explanatory model. The predominance of cross‐sectional designs further restricts causal inference and the understanding of developmental trajectories.

In response to these challenges, this study proposes the Differential Model of Giftedness and Talent, an integrative theoretical framework that seeks to clarify the distinction between cognitive potential and high‐level performance. In this model, giftedness is conceptualized as elevated cognitive potential—primarily associated with high general intellectual functioning and measurable through psychometric indicators—whereas talent represents the developmental outcome emerging from the progressive transformation of this potential into domain‐specific expertise. Psychological factors such as motivation, creativity, engagement and persistence function as key mechanisms that support both the expression of gifted potential and the long‐term development of talent.

By integrating psychometric evidence, developmental perspectives and findings from cognitive neuroscience, the proposed model aims to reduce conceptual ambiguity and provide a more coherent theoretical foundation for future research, identification practices and educational interventions involving gifted and high‐ability individuals. Future studies—particularly longitudinal and multimodal investigations combining psychometric and neurophysiological measures—will be essential for refining this framework and further elucidating the neurocognitive mechanisms underlying giftedness and talent development.

## Funding

The authors have nothing to report.

## Conflicts of Interest

The authors declare no conflicts of interest.

## Data Availability

Data sharing is not applicable to this article as no datasets were generated or analysed during the current study.
